# Working Smarter: Work-Related Emotional Intelligence and the Family-Work Interface

**DOI:** 10.3390/jintelligence13050058

**Published:** 2025-05-21

**Authors:** Michael D. Robinson, Kelyn X. Chen, Sukumarakurup Krishnakumar, Roberta L. Irvin

**Affiliations:** 1 Department of Psychology, North Dakota State University, Fargo, ND 58105, USA; roberta.irvin@ndsu.edu; 2 Rutgers New Jersey Medical School, Newark, NJ 07103, USA; kelynchen0@gmail.com; 3 Department of Management, University of Nevada, Las Vegas, NV 89154, USA; krishna.kumar@unlv.edu

**Keywords:** emotional intelligence, family-work conflict, work-related stressor outcomes

## Abstract

Employees have both work and non-work lives, and these domains of investment can interfere with each other. The present investigation (total *N* = 497) sought to understand the potential role(s) of work-related emotional intelligence (W-EI) in managing these forms of conflict, with samples consisting of full-time military personnel (Study 1), postdoctoral researchers (Study 2), and employees from diverse occupations (Study 3). Higher levels of W-EI were associated with lower levels of family-to-work conflict, but not work-to-family conflict, suggesting an asymmetric form of conflict shielding. Lesser experiences of family-work conflict also provided some explanation for why employees with higher W-EI levels were less prone to counterproductive work behaviors and work-related burnout. In addition, employees with higher W-EI levels were less prone to counterproductive work behaviors even when levels of family-work conflict were relatively high. The results highlight multiple ways in which employees with high W-EI levels manage the family-work interface.

## 1. Introduction

Individual differences figure prominently in the workplace (Sackett et al. 2017), and there has been considerable interest in the question of whether individual differences in emotional intelligence (EI) may have functional significance in this context (Ybarra et al. 2014). The answer to this question has been controversial, and results have often been disappointing (Miners et al. 2018; Robinson 2024; Ybarra et al. 2014), despite great interest in this construct within the corporate world (Caruso et al. 2016). There is some consensus that emotional intelligence should be assessed in ability-related terms (Daus and Love 2020; Roberts et al. 2010), but data involving ability-related tests (relative to self-report tests) are somewhat sparse, and findings have not been particularly encouraging (Miners et al. 2018; Ybarra et al. 2014). For example, in a meta-analysis, Miao et al. (2017b) found no relationship between ability EI levels and the frequency with which employees engaged in counterproductive work behavior. Because individuals with higher levels of EI should be better able to control their impulsive responses to emotion (Gratz and Tull 2010), such results might be considered puzzling.

In attempting to resolve gaps between theory and data, Krishnakumar et al. (2016) noted that the most common measures of ability EI—such as the MSCEIT (Mayer et al. 2003)—were not designed for workplace contexts. Indeed, the MSCEIT includes fairly abstract tasks (such as inferring the emotions present in paintings or likening emotion words to physical sensations) that may have little relevance to workplace contexts. Krishnakumar et al. (2016) therefore created an ability EI test that required individuals to make emotion-related judgments pertinent to the workplace context, specifically through the use of the situational judgment test method (Libbrecht and Lievens 2012). That is, the stems for the items all referenced hypothetical workplace events (or situations), and the events described were intended to be representative of most workplaces as well as consequential (e.g., how one might feel if layoffs were happening or if one encountered a struggling coworker). Personality-based measures contextualized for the workplace have been shown to predict workplace outcomes better than non-contextualized measures do (e.g., Bowling and Burns 2010; for a meta-analysis, see Shaffer and Postlethwaite 2012), and a work-based EI measure may similarly possess greater value in understanding individual differences in workplace functioning.

A number of investigations have provided support for this perspective in that employees with higher levels of work-related emotional intelligence (W-EI) appear to be better employees according to extant data. They appear to perform their job duties more effectively (Krishnakumar et al. 2016) and receive more commendations as a consequence (Krishnakumar et al. 2019a). They engage in organizational citizenship behaviors more frequently, and these associations remain significant when controlling for cognitive ability and personality (Robinson et al. 2023). They (those with higher W-EI levels) favor constructive conflict management strategies such as communicating, compromising, and problem-solving over forcing and yielding (Krishnakumar et al. 2019b). And they are less prone to deviant workplace behaviors of both interpersonal and organizational types (Robinson et al. 2019). Of note, the latter relationships also remain significant when controlling for personality and/or cognitive ability (Robinson et al. 2019), and there is some evidence for the idea that high W-EI employees, relative to low W-EI employees, are more capable of downregulating the pernicious influences of their negative affective states within the workplace (Krishnakumar et al. 2017).

Whether and how W-EI might matter within the context of work-family conflicts is not known, and this is an important area of study (Allen et al. 2014). All individuals are thought to invest in both work and non-work (e.g., family, leisure) roles, and these roles can often be incompatible with each other (Greenhaus and Beutell 1985). Workplace demands can make it difficult to accomplish one’s non-work goals, and family demands can interfere with one’s performance at work (Byron 2005). Generally speaking, conflicts of this type are considered stressors (Michel et al. 2011), and they can undermine job satisfaction, life satisfaction, and organizational commitment; in addition, they can increase burnout and give rise to turnover intentions (Netemeyer et al. 1996). Individuals handle family-work conflicts differently (Kossek and Lautsch 2012), and there are fascinating analyses of different work-family balance styles that emerge from considerations related to identity, behaviors, and attempts at boundary control (Kossek et al. 2012). Qualitative analyses are common in this area of research (Kossek et al. 2012), but there are also individual differences—such as negative affect or neuroticism—that systematically predict occurrences of work-family conflict as well as reactions to it (Allen et al. 2012).

Much of the original work in this area considered work-family conflict in monolithic terms (e.g., Wiley 1987), but it is useful to distinguish directions of influence. Although work-family (i.e., when work responsibilities interfere with family life) and family-work (i.e., when family and non-work matters interfere with attention to one’s job requirements) conflicts are positively correlated, this correlation is a moderate one (Netemeyer et al. 1996), and the two forms of conflict have different antecedents (Michel et al. 2011) and consequences (Kelloway et al. 1999). Individuals can be strategic in managing these directions of influence (Kossek and Lautsch 2012), and it is thought that greater investment in one’s workplace role might reduce occurrences of family-work conflict to a greater extent than occurrences of work-family conflict (Kossek et al. 2012). Central to the construct of work-related emotional intelligence is the idea that higher levels of it are linked to strategies that maximize workplace performance, in particular (Krishnakumar et al. 2016, 2019b). On the basis of this reasoning, we pursued the hypothesis that higher levels of W-EI would be inversely linked to experiences of family-work conflict, but not necessarily to experiences of work-family conflict. Indeed, fully investing oneself at work could sometimes result in higher, rather than lower, levels of work-family conflict (Michel et al. 2011). Though we did not predict positive correlations of this type, we did predict an asymmetry, and this asymmetry was investigated in two of the present studies.

To the extent that individuals higher in W-EI are better able to manage family-work conflicts, they should be less prone to some of the work-related strains that can follow from family-work conflict. According to stressor-strain theories (Radzali et al. 2013) and emotion-centered analyses (Shockley et al. 2012), this would include counterproductive work behaviors (CWBs), which are often conceptualized in terms of reactions to unwanted stressors (Spector 2011). Over longer periods of time, too, family-work conflict can lead to experiences of burnout within the workplace (Burke and Greenglass 2001). In the present studies, we examine potential relationships between W-EI and these strain-related outcomes. We also examine the important question of whether links between W-EI and these outcomes are at least partially explained by (i.e., mediated by) lesser tendencies toward family-work conflict. As others do (e.g., Côté 2014; Miners et al. 2018), we suggest that mediational analyses are critical in understanding the pathways and mechanisms that might link ability-related variations in emotional intelligence to key organizational outcomes.

In addition to examining questions of mediation, the present studies also examine questions of moderation. Stressors often generate negative emotions, and negative emotions can give rise to behaviors of an impulsive type (Carver et al. 2009; Frijda 2010). For example, feelings such as anger and irritation can trigger forms of aggression that are reactive and impulsive (Berkowitz 1983). Individuals who do not understand their emotions should be particularly vulnerable to such reflexive influences (Gratz and Tull 2010). By contrast, individuals who understand emotions and how they work should be capable of controlling their behaviors under conditions of emotional arousal (Peña-Sarrionandia et al. 2015). Such considerations suggest a moderation-related model whereby the influence of stressors on behavior should be stronger at lower levels of emotional intelligence than at higher levels of emotional intelligence (Krishnakumar et al. 2017). We pursue this hypothesis in the context of family-work conflict, proposing that it should engender higher levels of counterproductive work behavior, particularly at lower levels of W-EI.

Such behavioral control processes would be less relevant to work-related burnout, which is an experience rather than a behavior (Maslach et al. 2001). Based on such considerations, in addition to the idea that burnout is essentially uncontrollable given certain work and non-work conditions (Maslach 2003), we would not necessarily expect the relationship between family-work conflict and burnout to vary across levels of W-EI. By simultaneously examining potential moderating influences with respect to CWBs as well as burnout, we hoped to gain new knowledge concerning the scope of operations encompassed by the W-EI construct.

In the context of the ideas discussed above, we conducted a three-study program of research that examined the multiple ways in which ability-related emotional intelligence might interact with phenomena related to work-family conflicts. Individuals with higher levels of W-EI were expected to experience lower levels of family-to-work conflict, but not work-to-family conflict. Lesser tendencies toward family-work conflict were further hypothesized to mediate relationships between W-EI and two strain-related outcomes. Additionally, though, W-EI should moderate relationships between experiences of family-work conflict and tendencies toward CWBs, but not burnout. All studies focused on full-time employees, either within specific occupational niches (Studies 1 and 2) or with respect to a variety of occupations (Study 3).

## 2. Study 1

In Study 1, we sought to recruit a sample that should be particularly vulnerable to work-family conflicts. In this connection, Vuga and Juvan (2013) contend that issues of work-family balance are particularly difficult to navigate among military employees, who are also subject to numerous occupational stressors that many civilian employees do not experience, such as deployment (Campbell and Nobel 2009). Results involving military employees should, at the very least, provide some initial insights concerning the relationships of interest.

## 3. Method

### 3.1. Sample and Its Recruitment

We partnered with Qualtrics, which is a worldwide leader in research support provision. Qualtrics was charged with the specific task of recruiting 150 full-time active-duty United States military personnel from any branch other than the National Guard. As part of this recruitment effort, Qualtrics invited 620 qualified individuals, 297 of whom started but did not necessarily finish the survey, and 164 of whom finished the survey while passing attention checks. Twelve of these individuals were deemed to have completed the survey too quickly, resulting in a final sample size of 152.

All respondents had completed high school, and 54% of them had completed some college classes or had obtained two- or four-year college degrees. The majority of respondents (76.97%) were married. The sample was, however, diverse with respect to age (*M* = 31.95; *SD* = 8.27), sex (40.13% female), ethnicity (71.05% White, 14.47% Black, 6.58% Hispanic, 3.95% Asian, 3.95% other), geographic location (participants were stationed in 26 U.S. states), job description (e.g., air traffic controller, electronics technician, military police, registered nurse), and military branch (44.08% army, 25.66% air force, 24.34% navy, 5.92% marines). The average respondent had served in the military for 112.25 months (*SD* = 93.38).

The study was programmed with Qualtrics software and administered online. After reporting on demographic information, respondents completed the predictor and outcome measures described below. Because interactions involving sex were not significant (*p*s > 0.200), this variable is omitted from further consideration. Of note, results involving the Study 1 sample are reported in BLINDED, but the current paper focuses on an entirely different set of process and outcome variables. Data for this project are available at https://osf.io/waqxr/?view_only=a6c39790eaf545d2b355de1598f543d7 (accessed on 1 May 2025). Informed consent was obtained from all participants in all studies, which were also approved by local IRBs. Below, we will introduce the key measures, which are work-related emotional intelligence, work-family and family-work conflict, counterproductive work behavior, and work-related burnout.

### 3.2. Work-Related Emotional Intelligence

To understand manifestations of emotional intelligence within the workplace, Krishnakumar et al. (2016) created an ability-based measure particularly suited to this context. The test, which measures what is termed work-related emotional intelligence (W-EI), uses the situational judgment platform (Corstjens et al. 2017) to probe for emotional inferences as well as ideas about effective ways of responding (McDaniel et al. 2007) to a series of scenarios involving workplace events possessing emotional significance (e.g., in relation to challenges in meeting deadlines or interpersonal disagreements). Krishnakumar et al. (2016) amassed considerable evidence in support of the reliability and validity of the test, and subsequent studies have highlighted how the relevant skills matter for workplace experience (e.g., Robinson et al. 2020), behavior (e.g., Robinson et al. 2019), and performance (e.g., Krishnakumar et al. 2019a). The test correlates with other measures of ability EI (Krishnakumar et al. 2016), but is relatively unique in its focus on work-contextualized inferences and abilities (Robinson et al. 2019).

In concrete terms, participants responded to 20 scenarios (e.g., “There have been widespread layoffs in Margie’s organization recently”). After reading 10 of them, participants were asked to rate the extent to which (1 not at all; 5 = very strongly) the protagonist (in this case, Margie) would experience each of 4 emotions (anxiety, anger, fear, confusion). After reading the other 10 scenarios (e.g., “Jake and his employees are rushing toward a tight deadline. Unfortunately, the computers crash while attempting to meet the deadline”.), participants were asked to rate the effectiveness (1 = not at all effective; 5 = very effective) of 4 ways of responding to the described situation (e.g., “inform the company that the deadline cannot be met”, “scold the employees for not having backed up the computer”). All 80 ratings (20 scenarios times 4 responses) were then rescored in terms of the percentage of an expert sample of 82 business leaders, with an average of 18.53 years of work experience and an average of 27.15 supervisees, who gave the same rating for the particular scenario/response pairing (e.g., if the participant made a 4 rating and 31.71% of the expert sample also made a 4 rating for that particular scenario/response pairing, the participant would receive a score of 0.3171). After scoring responses in this manner, each participant received a W-EI score that averaged across responses for a particular scenario and then across scenarios (*M* = 0.3324; *SD* = 0.0458; α = 0.87). This combined score is thought to reflect both explicit and implicit (or behavioral) emotion knowledge (Robinson et al. 2020), as applied to workplace settings (Robinson et al. 2019).

### 3.3. Work-Family and Family-Work Conflict

Given that individuals have both work and non-work lives, events and conditions from one sphere can interfere with the other (Kossek and Lautsch 2012), and both family-to-work as well as work-to-family conflicts are possible (Allen et al. 2014). To investigate both forms of conflict in a separable manner, we administered the work-to-family conflict (WFC) and family-to-work conflict (FWC) scales of Netemeyer et al. (1996). As is commonly observed (Ford et al. 2007), participants reported higher levels of WFC (*M* = 3.98; *SD* = 1.78; α = 0.95) relative to FWC (*M* = 2.55; *SD* = 1.39; α = 0.93), but family-work conflict, by definition at least, could often be more problematic to workplace functioning (Kossek and Lautsch 2012). Individuals with high, relative to low, levels of W-EI may be better capable of mitigating these influences.

### 3.4. Counterproductive Work Behavior

The focus of the present investigation was on problematic—or strain-related (Fox et al. 2001)—responses to family-work conflicts. As one potential strain of this type, we assessed tendencies toward counterproductive work behavior (CWB), which employees may typically engage in when they are stressed, frustrated, or angry (Spector 2011). Participants indicated how frequently (1 = never; 5 = everyday) they engaged in counterproductive acts such as purposely wasting the employer’s materials or insulting others concerning their job performance in relation to the 10-item version (Spector et al. 2010) of the CWB checklist (α = 0.88). It is useful to note that self-reports of CWB tend to be more valid than coworker or supervisor reports of CWB (Berry et al. 2012), typically because many CWBs are concealed from employers and/or are difficult to observe (Carpenter et al. 2017).

### 3.5. Work-Related Burnout

Chronic stressors can lead to workplace burnout, defined in terms of a state of exhaustion within the workplace that is inimical to job satisfaction and performance (Maslach et al. 2001). We assessed such experiences using the work-related burnout scale of the Copenhagen Burnout Inventory (Kristensen et al. 2005), which has performed well in a number of prior studies in a number of cultures (e.g., Fong et al. 2014). The scale was scored such that higher numbers reflected higher levels of burnout (α = 0.89).

## 4. Results

### 4.1. Zero-Order Correlations

Table 1 provides descriptive statistics for the key variables and reports zero-order correlations among the variables. The family-work and work-family conflict scales were positively correlated, but the magnitude of the correlation did not approach unity. Of particular interest, individuals obtaining higher W-EI scores reported lower levels of family-work conflict (FWC), but not lower levels of work-family conflict (WFC). Thus, W-EI seems to protect against spillover from the family domain to the work domain more than it protects against spillover from the work domain to the family domain. As expected, higher levels of family-work conflict were linked to higher levels of CWB as well as burnout. In zero-order terms, W-EI was not predictive of CWB frequencies or burnout.

### 4.2. Mediation Through Family-Work Conflict

Work-related emotional intelligence was protective against family-work conflict, and family-work conflict predicted both counterproductive work behaviors and burnout. It is therefore feasible that there are indirect relationships involving FWC that link W-EI to each of the outcomes (MacKinnon and Fairchild 2009). To investigate this possibility, we performed two mediation-related analyses, one for each strain-related outcome. In both cases, we used the SAS 9.4-based PROCESS macro created by Hayes (2013), and all variables were z-scored to aid magnitude interpretation (Krishnakumar and Robinson 2015).

**Figure 1:**
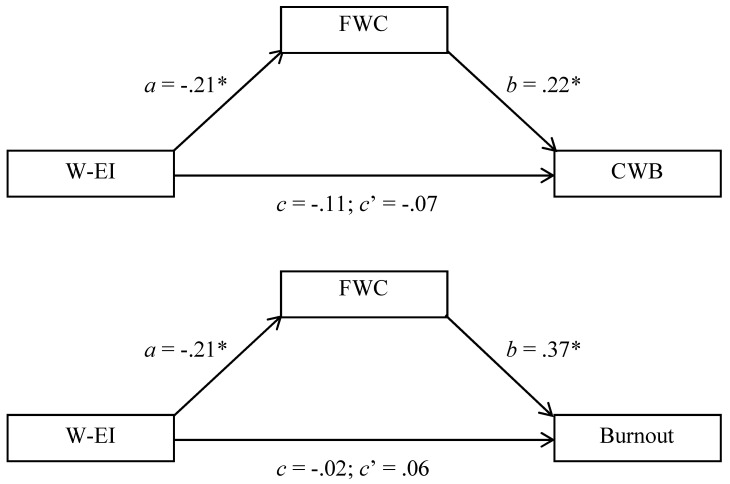
Family-Work Conflict (FWC) as a Mediator Linking Work-Related Emotional Intelligence (W-EI) to Counterproductive Work Behavior (CWB: **Top** Panel) and Workplace Burnout (Burnout: **Bottom** Panel), Study 1. * *p* < 0.05.

Figure 1 reports key results for these analyses. Of importance, direct effects (*c’*) were smaller than total effects (*c*), or of opposite signs, indicating the possibility of mediation (MacKinnon and Fairchild 2009). Whether mediational pathways were significant was determined by calculating 95% bias-corrected confidence intervals (BCCIs) for the indirect (*ab*) pathways (Hayes 2013). These confidence intervals excluded 0 for both the CWB, BCCI = −0.142 to −0.004, and burnout, BCCI = −0.177 to −0.017, outcomes, indicating that both mediational pathways were significant. In the CWB model, the indirect pathway accounted for 39.94% of the variance linking W-EI to CWB; in the burnout model, this estimate was 453.53%, given the reversal of sign. What these numbers suggest is that lesser tendencies toward FWC are substantially implicated in the protective effects of W-EI. Nonetheless, we do admit that these results are not fully compelling, in part because zero-order relationships linking W-EI to the outcomes were not significant. We will therefore revisit these mediational pathways in two additional studies.

### 4.3. Results Involving Moderation

**Figure 2:**
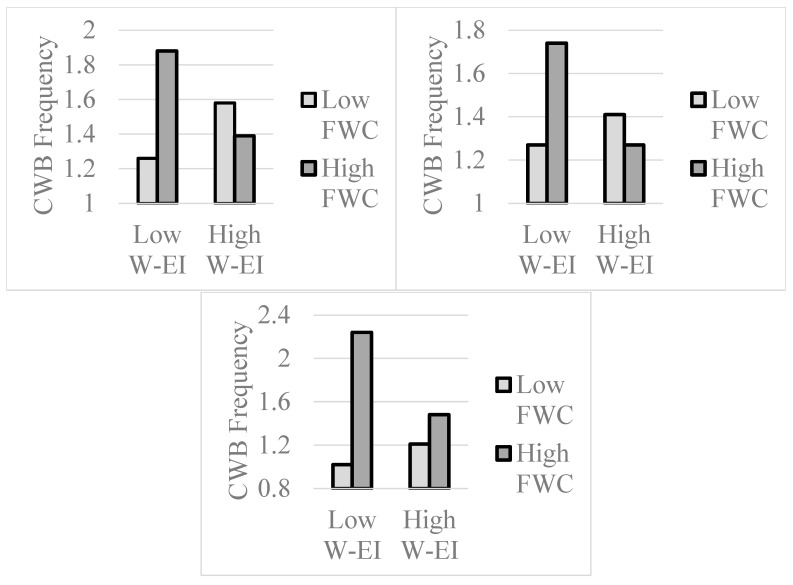
Work-Related Emotional Intelligence (W-EI) and Family-Work Conflict (FWC) Interact to Predict Counterproductive Work Behaviors (CWB), Studies 1 (**Top Left** Panel), 2 (**Top Right** Panel), and 3 (**Bottom** Panel).

We next examined whether family-work conflict was less problematic at higher levels of W-EI. In these moderation-related multiple regressions, we entered z-scored versions of W-EI and FWC, as well as their interaction term, as predictors of each of the strain-related outcomes (Aiken and West 1991). When predicting CWB frequencies, there was a main effect for FWC, *t* = 2.33, *p* = 0.021, β = 0.18, no main effect for W-EI, *t* = 0.90, *p* = 0.370, β = 0.07, but the W-EI by FWC interaction was significant, *t* = −4.24, *p* < 0.001, β = −0.34. Estimated means (+1/−1 *SD*) for this interaction are displayed in the top left panel of Figure 2, and simple slopes analyses (Aiken and West 1991) indicated that the FWC/CWB relationship was significant at low (−1 *SD*) levels of W-EI, *t* = 4.98, *p* < 0.001, β = 0.52, but not at high (+1 *SD*) levels of W-EI, *t* = −1.36, *p* = 0.177, β = −0.16. These results suggest that high W-EI individuals are capable of controlling their counterproductive behaviors when experiencing higher levels of family-work conflict.

In the parallel analysis of burnout, the main effect for FWC remained significant, *t* = 4.82, *p* < 0.001, β = 0.38, but there was no main effect for W-EI, *t* = 0.77, *p* = 0.442, β = 0.06, and there was no W-EI by FWC interaction, *t* = 0.97, *p* = 0.334, β = 0.08. Hence, even though high W-EI individuals with high levels of family-work conflict were capable of controlling their tendencies toward counterproductive behavior, they were not capable of controlling their experiences of work-related burnout. This is likely because behaviors are more controllable than experiences of exhaustion (Maslach 2003).

## 5. Discussion and Study 2

The results of Study 1 provide initial evidence for three key findings: (1) W-EI protects against family-to-work conflicts, (2) lesser family-work conflict mediates at least a portion of the variance linking W-EI to strain-related outcomes, and (3) W-EI buffers the effect of family-work conflict on counterproductive work behavior. For the sake of generalizability, it seemed important to replicate these findings within a different occupational context, particularly given conditions in the military that are not present in other jobs (Campbell and Nobel 2009). Study 2, therefore, focuses on the same relationships among a sample of postdoctoral researchers. The postdoctoral position is another one linked to high levels of stress. Many postdoctoral employees start new families but work in positions whose capacity to serve as stepping stones for an academic career is in doubt (Åkerlind 2005). Postdoctoral researchers also work long hours in positions that can be frustrating due to insufficient resources, inconsistent supervision, and a lack of institutional support (Chen et al. 2015). As a result of conflicting demands on their time, postdoctoral employees can struggle to achieve a work-life balance (Åkerlind 2005), rendering this sort of sample a useful one in examining the processes of interest.

## 6. Method

### 6.1. Sample and Its Recruitment

We recruited a sample of postdoctoral researchers in two ways. Initially, a research assistant gathered contact information for 1076 postdoctoral researchers—in programs such as biology, biochemistry, wildlife biology, and ecology—who were listed on university websites throughout the United States. This recruitment strategy was supplemented by a second one that involved sending an email to the listserv of the National Postdoctoral Association, which reaches approximately 3400 postdoctoral researchers within both industry and university settings. In both cases, potential responders were informed that we were conducting an IRB-approved study on postdoctoral experiences and that 1 in 10 responders would receive a $20 gift certificate. In both cases, also, a link to a secure Qualtrics-programmed survey was also provided. The first mode of recruitment resulted in 40 completed surveys, and the second resulted in 158, yielding a total of 198 participants.

All participants had received PhDs or other comparable degrees. The average age was 32.76 (*SD* = 4.68), and 62.46% of the responders were female. The sample was diverse with respect to ethnicity (59.73% White, 29.01% Asian, 4.44% Hispanic, 3.41% Pacific Islander, 3.07% Black, 0.34% Native American), field of study, and research settings (e.g., university, medical centers). The majority of the sample was married or engaged (60.06%), while others were separated (0.34%), divorced (2.39%), or single (37.2%). Even among single individuals, however, family-work conflicts can occur when family is broadened to include unmarried partners, parents, or close friends (Kossek and Lautsch 2012). The average tenure in the current position was 24.21 months, but some researchers had much longer tenures (*SD* = 31.15 months), which is consistent with the idea that the postdoctoral position can become a sort of limbo position on the way to a career (Åkerlind 2005). The average annual salary was approximately $46,000.

The Qualtrics-programmed survey was administered online. Participants first completed the demographic measures, then the emotional intelligence task. Subsequently, they completed the family-work conflict and outcome measures. Some results involving this sample are reported in BLINDED, but the focus on family-work conflict experiences is unique to the current report. As in Study 1, the key variables, which will be introduced next, are work-related emotional intelligence, work-family and family-work conflict, counterproductive work behavior, and work-related burnout.

### 6.2. Work-Related Emotional Intelligence

Work-related emotional intelligence was assessed in the same manner as in Study 1, and descriptive statistics were comparable to the first study (*M* = 0.3194; *SD* = 0.0469; α = 0.86).

### 6.3. Work-Family and Family-Work Conflict

Participants reported on family-to-work (e.g., “Family-related strain interferences with my ability to perform job-related duties”) and work-to-family (e.g., “The demands of my work interfere with my home and family life”) conflicts using the Netemeyer et al. (1996) scales, also used in Study 1. As in Study 1, the WFC mean (*M* = 4.13; *SD* = 1.69; α = 0.96) was higher than the FWC mean (*M* = 2.79; *SD* = 1.46; *α* = 0.92), but higher levels of W-EI may be particularly protective against family-work conflict, given W-EI’s emphasis on competent workplace functioning (Krishnakumar et al. 2016).

### 6.4. Counterproductive Work Behavior

Participants indicated how frequently they engaged in 10 counterproductive workplace behaviors (e.g., “purposely wasted your employer’s materials/supplies”) using the same Spector et al. (2010) measure used in Study 1. Tendencies toward these behaviors were reliable across items, and we therefore computed a CWB total score (α = 0.89).

### 6.5. Work-Related Burnout

Experiences of work-related burnout were assessed using the pertinent scale of Kristensen et al. (2005), which was also used in Study 1 (α = 0.87).

## 7. Results

### 7.1. Zero-Order Correlations

As displayed in Table 2, the WFC and FWC scales were positively correlated. Even so, W-EI was protective against family-to-work conflict but not work-to-family conflict. We therefore concentrate on the FWC scale in additional analyses. In Study 2, W-EI was protective against both counterproductive work behaviors and (to a lesser degree) burnout. Also of importance, individuals who reported higher levels of FWC were prone to both counterproductive work behaviors and burnout, suggesting the possibility of mediation-related pathways.

### 7.2. Mediation Through Family-Work Conflict

**Figure 3:**
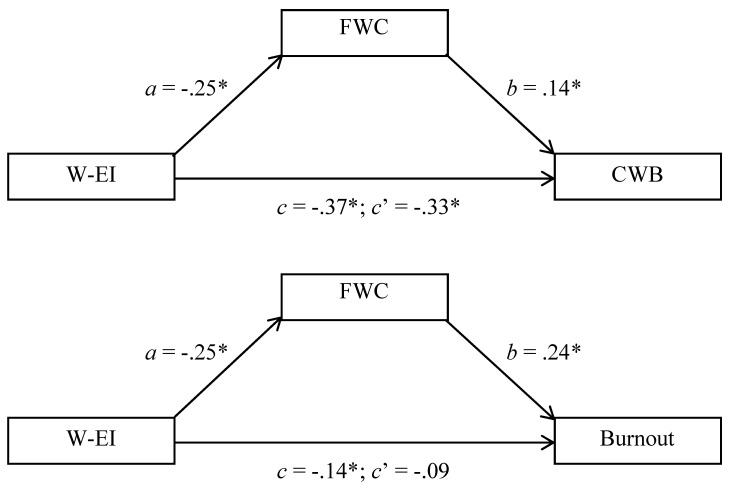
Family-Work Conflict (FWC) as a Mediator Linking Work-Related Emotional Intelligence (W-EI) to Counterproductive Work Behavior (CWB: **Top** Panel) and Workplace Burnout (Burnout: **Bottom** Panel), Study 2. * *p* < 0.05.

Mediation analyses were conducted in a manner parallel to Study 1, and Figure 3 reports key results for these analyses. In both cases, direct effects (*c*’) were smaller than total effects (*c*), suggesting the possibility of mediation (MacKinnon and Fairchild 2009). Whether the mediational pathways were significant was determined by calculating 95% bias-correlated confidence intervals for indirect (*ab*) pathways. These confidence intervals excluded 0 for both the CWB, BCCI = −0.083 to −0.009, and burnout, BCCI = −0.111 to −0.022, outcomes, indicating significant mediation in both cases (Hayes 2013). A comparison of the mediational and total pathways indicated that 39.92% of the W-EI/burnout relationship could be attributed to family-work conflict, and this figure was a more meager 9.55% with respect to the W-EI/CWB relationship. A moderation-related model might possess greater explanatory value in the latter case.

### 7.3. Results Involving Moderation

A second set of analyses focused on whether W-EI moderated the impact of family-work conflict. In these multiple regressions, z-scored versions of W-EI and FWC, as well as a W-EI by FWC interaction term, were entered as predictors of each of the outcomes (Aiken and West 1991). In the analysis predicting CWB frequencies, main effects for W-EI, *t* = −4.19, *p* < 0.001, β = −0.28, and FWC, *t* = 2.35, *p* = 0.020, β = 0.15, were observed, and there was also a W-EI by FWC interaction, *t* = −2.37, *p* = 0.018, β = −0.15. Estimated means (+1/−1 *SD*) for the interaction are displayed in the top right panel of Figure 2 (see above), and simple slopes analyses revealed that FWC was a predictor of CWB at low (−1 *SD*) levels of W-EI, *t* = 3.27, *p* = 0.001, β = 0.30, but not at high (+1 *SD*) levels of W-EI, *t* = 0.03, *p* = 0.979, β = 0.00. These results replicate Study 1 and suggest that high W-EI individuals are capable of mitigating the behavioral consequences of higher levels of family-work conflict.

In the parallel analysis of burnout, the FWC main effect was significant, *t* = 3.55, *p* < 0.001, β = 0.24, but the main effect for W-EI was not significant, *t* = −1.29, *p* = 0.200, β = −0.09, and the W-EI by FWC interaction was also not significant, *t* = 0.30, *p* = 0.764, β = 0.02. Hence, and in parallel to Study 1, higher levels of family-work conflict were linked to higher levels of burnout among all individuals, irrespective of their W-EI levels.

## 8. Discussion and Study 3

Although military and postdoctoral positions are in many ways very different (Chen et al. 2015; Redmond et al. 2015), the results of Studies 1 and 2 largely converged. Still, it should be recognized that these are both highly particular occupations, and there are benefits to recruiting samples with more heterogeneous job descriptions and responsibilities (Greenidge and Coyne 2014). In Study 3, we sought to recruit a diverse sample of this type, such that results involving the sample might provide the most general conclusions concerning the processes of interest.

## 9. Method

### 9.1. Sample and Its Recruitment

Qualtrics, a leader in large-scale research services, was charged with recruiting 150 employees older than 23 who had full-time jobs in the United States. We also specified an even mix of males and females. Qualtrics announced the study opportunity, ensured that responders met the criteria, and deleted participants who failed any one of the 4 attention checks. Additionally, we deleted 2 individuals for responding too quickly, resulting in a final sample size of 147.

The average age of responders was 42.78 (*SD* = 11.19). In addition, the sample was diverse with respect to gender (48.85% female), ethnicity (71.00% White, 12.98% Black, 9.16% Hispanic, 4.58% Asian, 0.76% other), geographical location (42 of 50 states), and occupation (e.g., accountant, contractor, CEO, educator, janitor, machinist, nurse, police officer). A slight majority of individuals were married (54.62%), and the average job tenure was 11.34 years (*SD* = 8.60). The average annual salary among these employees was $61,078.

A Qualtrics-programmed survey was administered online, and the order of measures was similar to prior studies (e.g., demographics, then EI test, etc.). Results involving this sample have been reported elsewhere (e.g., BLINDED), but the current focus on family-work conflict experiences is unique to the current report. Next, we will describe the key measures for the study, which are work-related emotional intelligence, family-work conflict, counterproductive work behavior, and work-related burnout.

### 9.2. Work-Related Emotional Intelligence

Work-related emotional intelligence was assessed in a manner identical to prior studies (*M* = 0.3175; *SD* = 0.0503; α = 0.89).

### 9.3. Family-Work Conflict

Given the results of prior studies, we administered Netemeyer et al.’s (1996) family-to-work conflict scale (α = 0.96), but not their WFC scale.

### 9.4. Counterproductive Work Behavior

Employees reported on their CWB frequencies using the Spector et al. (2010) measure, also used in prior studies (α = 0.96).

### 9.5. Work-Related Burnout

Experiences of work-related burnout were assessed in a manner identical to prior studies (α = 0.88).

## 10. Results

### 10.1. Zero-Order Correlations

Table 3 reports remaining descriptive statistics as well as correlations among the variables. As in prior studies, W-EI was protective against family-to-work conflict. In addition, W-EI displayed inverse relationships with CWB frequencies and (to a lesser extent) burnout. Finally, higher levels of family-work conflict were associated with higher CWB frequencies as well as with more pronounced experiences of burnout.

### 10.2. Mediation Through Family-Work Conflict

**Figure 4:**
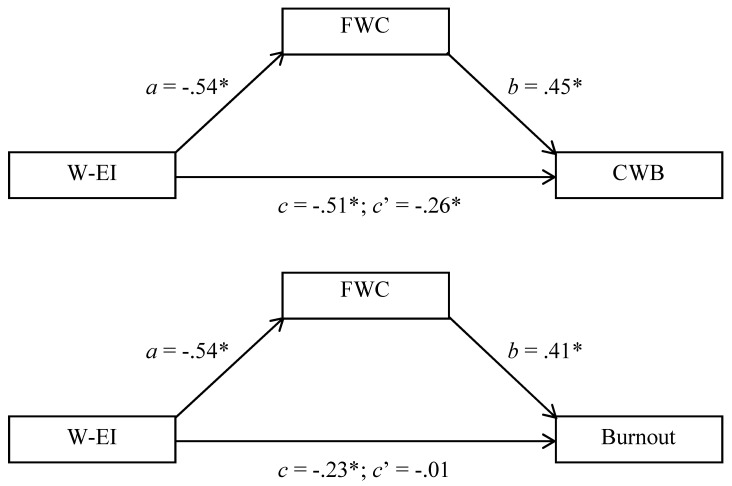
Family-Work Conflict (FWC) as a Mediator Linking Work-Related Emotional Intelligence (W-EI) to Counterproductive Work Behavior (CWB: **Top** Panel) and Workplace Burnout (Burnout: **Bottom** Panel), Study 3. * *p* < 0.05.

Mediation analyses were conducted in a manner parallel to prior studies, and Figure 4 reports key results for these analyses. In both cases, direct effects (*c*’) were smaller than total effects (*c*), suggesting the possibility of mediation. The mediation pathway for the CWB model was significant, BCCI = −0.397 to −0.141, and the mediational pathway for the burnout model was also significant, BCCI = −0.350 to −0.122. The mediational pathway (relative to the total pathway) accounted for 48.57% of the variance linking W-EI to CWB (first model) and 97.32% of the variance linking W-EI to burnout (second model).

### 10.3. Results Involving Moderation

As in prior studies, we turned to the question of whether W-EI moderates the strain-related impact of FWC. In these regression models, z-scored versions of W-EI and FWC, as well as their interaction term, were entered as predictors of each of the outcomes. In the analysis predicting CWB frequencies, main effects for W-EI, *t* = −2.07, *p* = 0.040, β = −0.16, and FWC, *t* = 5.62, *p* < 0.001, β = 0.41, were both significant. In addition, there was a significant W-EI by FWC interaction, *t* = −3.86, *p* < 0.001, β = −0.26, and estimated means for this interaction are displayed in the bottom panel of Figure 2. As suggested by the figure, the FWC/CWB relationship was significant at low (−1 *SD*) levels of W-EI, *t* = 7.27, *p* < 0.001, β = 0.68, but not at high (+1 *SD*) levels of W-EI, *t* = 1.43, *p* = 0.154, β = 0.15.

In the parallel analysis concerning burnout, the main effect for FWC was significant, *t* = 4.66, *p* < 0.001, β = 0.42. Consistent with the mediation-related results reported above, W-EI was not a significant predictor of burnout when controlling for FWC, *t* = −0.52, *p* = 0.601, β = −0.05. Finally, there was no W-EI by FWC interaction, *t* = 1.39, *p* = 0.166, β = 0.116, indicating that family-work conflict was equally predictive of burnout at high as well as low levels of work-related emotional intelligence.

## 11. Discussion

Relative to Studies 1 and 2, we used a broader sampling strategy in Study 3, and the findings were particularly robust. Employees with higher levels of work-related emotional intelligence were less prone to family-work interference or conflict, and this relationship was consequential in linking W-EI to both of the strain-related outcomes. In fact, the relationship between W-EI and burnout, in Study 1, was almost fully attributed to the protective role that W-EI plays in mitigating family-work conflict. Although family-work conflict also mediated the relationship linking W-EI to counterproductive work behaviors, the more remarkable pattern involved an interaction such that family-work conflict seems to precipitate CWBs exclusively among individuals possessing lower levels of ability-related EI. Although the findings of Study 3 were particularly strong, the findings from all three studies largely converged in highlighting family-work conflict as an important nexus through which variations in W-EI operate.

## 12. General Discussion

Conflicts between work and non-work domains are almost inevitable (Greenhaus and Beutell 1985), and the goal of the present investigation was to examine how individuals with varying levels of work-related emotional intelligence (W-EI) negotiate the work/non-work interface. To provide convincing data concerning such questions, we examined the same relationships across multiple studies, all of which involved full-time workers, either within particular occupations (Studies 1 and 2) or from a broader range of occupations (Study 3). By holding key measures constant, the goal was to obtain new, replicable insights concerning the processes of interest.

The findings indicate that employees with higher levels of W-EI shield the work domain, such that issues related to family and non-work lives are less likely to interfere with responsibilities at work (correlations between W-EI and family-to-work conflict were −0.21, −0.24, and −0.54 in Studies 1, 2, and 3, respectively). This shielding can be considered asymmetric (Allen et al. 2014); however, in that the same individual differences were not protective against work-to-family conflict (these correlations were −0.03 and 0.01 in Studies 1 and 2). The asymmetric correlations, therefore, implicate strategic factors that should function to preserve workplace performance, or at least workplace investment, at higher levels of W-EI.

Evidence in support of this perspective was obtained by linking family-work conflict to two strain-related outcomes that can follow from it, namely, counterproductive work behaviors (Radzali et al. 2013) and burnout (Burke and Greenglass 2001). As hypothesized, both counterproductive work behaviors (correlations of 0.23, 0.22, and 0.59 in Studies 1, 2 and 3, respectively) and burnout (correlations of 0.36, 0.25, and 0.41) were positively linked to occurrences of family-work conflict and occurrences of family-work conflict also mediated relationships linking W-EI to each of these outcomes. Mediational pathways were stronger with respect to burnout (percentage of variance accounted for: 453.53%, 39.92%, and 97.32% in Studies 1, 2 and 3) than with respect to CWB frequencies (39.94%, 9.55%, and 48.57%), however, indicating that reduced occurrences of family-work conflict were particularly valuable in protecting against burnout among individuals possessing higher levels of W-EI.

Relative to burnout, though, counterproductive work behaviors are thought to be controllable, in principle (Spector 2011), and W-EI was protective in this context. Specifically, W-EI and family-work conflict levels consistently interacted to predict CWB frequencies. Furthermore, it was found that family-work conflict was linked to higher levels of CWB at low levels of work-related emotional intelligence, but not at high levels. Knowledge concerning emotions and how to handle them, then, was protective against CWBs even when levels of family-work conflict were relatively high. The findings, therefore, highlight both mediation- and moderation-related mechanisms linking W-EI to the effective management of family-work conflict, providing a nuanced perspective on this interface.

### 12.1. Further Implications

Organizational scholars have, at times, been enthusiastic about the ability EI construct (Daus and Love 2020), but findings in this area have not always been encouraging (Miners et al. 2018; Robinson 2024; Ybarra et al. 2014). With the creation of a reliable and valid measure of ability EI that targets workplace functioning (Krishnakumar et al. 2016) and the findings that subsequently accrued (e.g., Krishnakumar et al. 2019a; Robinson et al. 2019), we believe that skepticism concerning the value of EI in organizational contexts (e.g., Dasborough et al. 2022) needs to be revisited. Individuals who can make more accurate inferences concerning workplace emotions and who possess knowledge concerning appropriate ways of responding to emotionally evocative workplace situations appear to be better employees (e.g., Robinson et al. 2023), and the present findings provide hitherto unavailable support for this idea.

In this connection, it is customary to think that family stressors predispose individuals to family-work conflict, whereas work stressors predispose individuals to work-family conflict. However, individuals differ considerably in how these boundaries are managed (Allen et al. 2014), and some individuals may possess strategies that render it less likely that what happens outside of the workplace affects workplace functioning (Kossek and Lautsch 2012). The present results suggest that work-related emotional intelligence may be a critical variable of this type. It was linked to asymmetries in conflict, suggesting the down-regulation of family-to-work conflict in particular, and it was linked to lesser tendencies toward counterproductive work behavior even when family-work conflict was experienced. Thus, what is essentially a work-related variable (i.e., W-EI) appears to function in a manner that reduces cross-over from that which occurs outside of the workplace. Vice versa, the findings suggest that an ability EI measure that targets family settings might buffer work-to-family conflict more than it buffers family-to-work conflict. This exciting possibility requires additional research, which might also require the creation of an ability EI measure that concentrates on family-related events and circumstances.

There are various ways in which W-EI could function as it does. The skills and capacities encompassed by W-EI could bolster job resources, which tend to motivate job performance (Balducci et al. 2011). Consistent with this possibility, ability-related variations in EI have been linked to job resources in meta-analytic work (Miao et al. 2017a). It is also possible that individuals with higher levels of W-EI perceive that they have greater control over workplace stressors, and perceived control has been linked to proactive coping and more benign interpretations of workplace events and conditions (Spector 2002). It is also likely that W-EI supports workplace engagement, which is predictive of higher levels of organizational citizenship and lesser experiences of burnout, among other outcomes (Rich et al. 2010). In other words, the present findings encourage further research on the factors and perceptions that protect workplace performance among individuals with higher levels of W-EI.

Turning to a different point, the focus on two different strain-related outcomes provided insights into the scope of W-EI operations. Counterproductive work behavior is typically impulsive, and it is typically exhibited under conditions associated with frustration, stress, and irritation (Spector 2011). The present results suggest that individuals possessing higher levels of W-EI are capable of controlling their behaviors in the context of higher levels of stress, whereas individuals possessing lower levels of W-EI are less capable of doing so. These results link lower levels of W-EI to something like emotional impulsivity (Carver and Johnson 2018) or negative urgency (Cyders and Smith 2008), which gives rise to dysregulated behaviors of multiple types (Settles et al. 2012). Indeed, it makes a great deal of sense to propose that what ability EI consists of, in part, is a capacity to control one’s behaviors under conditions of emotional upset (Gratz and Tull 2010; Krishnakumar et al. 2017). This control does not, however, extend to experiences of burnout, which may be somewhat inevitable given stressful job conditions (Maslach 2003). Even so, W-EI could moderate burnout’s relationship with behavioral manifestations such as absenteeism or turnover (Kristensen et al. 2005). Moderation-related results of this type would be consistent with the interactions reported in Figure 2.

### 12.2. Questions, Limitations, and Future Directions

We did not conduct an extensive survey of family conditions, and it might be useful to do so in future research. Nonetheless, scholars in this area have concluded that nearly all employees—and even single employees—have family lives that they are invested in (Kossek and Lautsch 2012; Rothausen 1999). Hence, what the present results indicate is that all of these outside-of-work conditions, whichever they are, are perceived to interfere with workplace functioning to a lesser extent at higher levels of W-EI. This is impressive in part because W-EI is linked to social skills and prosocial functioning (e.g., Krishnakumar et al. 2019b), which would tend to be associated with broader social obligations outside of the workplace (Robinson et al. 2023).

The process and outcome measures were self-reported, and this was appropriate given the variables that were focused on. For example, the experience of work-related burnout is a subjective experience that individuals should be asked to report on themselves (Kristensen et al. 2005). Counterproductive behaviors were also self-reported, but this method has been recommended because self-reports of CWB appear to be more valid than other-reports of CWB (Berry et al. 2012), in part because many occurrences of CWB are hidden from others and/or are simply not very observable by anyone other than the self (Carpenter et al. 2017). It is also useful to note that W-EI was assessed in ability-related rather than self-reported terms, precluding issues related to mono-method correlations (Daus and Love 2020; Mayer et al. 2008, 2016).

The designs were cross-sectional in nature and could be supplemented with other designs in future research. For example, it might make sense to use longitudinal designs in future research, particularly in capturing effects that may build over time (Ford et al. 2014), as could be the case with experiences of work-related burnout (Kristensen et al. 2005). Additionally, recent research has suggested that quite a few variables possessing organizational relevance, such as family-work conflict (Nohe et al. 2014), wax and wane on a within-subject basis (Dalal et al. 2020). Experience-sampling designs could therefore be used in tracking some of the relationships that were observed in the present studies, as a complement to the present methods. Finally, the present results encourage efforts to train EI abilities within the workplace context. Recent meta-analyses have indicated that EI training is at least somewhat efficacious (Hodzic et al. 2018; Kotsou et al. 2019), and EI training that targets workplace settings and relationships might be particularly effective with respect to the sorts of outcomes that were focused on in the present research.

### 12.3. Conclusions

The present studies provide an in-depth analysis concerning the interface between work-related emotional intelligence (W-EI) and conflicts related to work-family balance. Work-related emotional intelligence was protective against family-work conflict, in particular terms, and this relationship was explanatory in linking W-EI to counterproductive work behaviors as well as to experiences of work-related burnout. Additional results indicated that W-EI moderated the extent to which family-work conflicts were linked to counterproductive work behavior, but not burnout. The research, therefore, highlights multiple mechanisms that protect against family-work conflict among employees possessing higher levels of W-EI.

## Figures and Tables

**Table 1:** Correlations among key variables, Study 1.

Variable	*M*	*SD*	1	2	3	4	5
1. W-EI	0.33	0.05	—				
2. FWC	2.55	1.39	−0.21 *	—			
3. WFC	3.98	1.78	−0.03	0.39 **	—		
4. CWB	1.57	0.59	−0.11	0.23 **	0.20 *	—	
5. Burnout	2.78	0.92	−0.02	0.36 **	0.52 **	0.41 **	—

Note: * *p* < 0.05; ** *p* < 0.01.

**Table 2:** Correlations among key variables, Study 2.

Variable	*M*	*SD*	1	2	3	4	5
1. W-EI	0.32	0.04	—				
2. FWC	2.78	1.43	−0.28 **	—			
3. WFC	4.06	1.71	−0.01	0.47 **	—		
4. CWB	1.44	0.55	−0.36 **	0.25 **	0.17 *	—	
5. Burnout	2.75	0.87	−0.19 **	0.31 **	0.52 **	0.20 *	—

Note: * *p* < 0.05; ** *p* < 0.01.

**Table 3:** Correlations among key variables, Study 3.

Variable	*M*	*SD*	1	2	3	4
1. W-EI	0.32	0.05	—			
2. FWC	2.77	1.81	−0.54 **	—		
3. CWB	1.61	0.90	−0.50 **	0.59 **	—	
4. Burnout	2.56	0.94	−0.23 **	0.41 **	0.39 **	—

Note: ** *p* < 0.01.

## Data Availability

Data for this project are available at https://osf.io/waqxr/?view_only=a6c39790eaf545d2b355de1598f543d7 (accessed on 1 May 2025).
